# Automatic processing of unattended object features by functional connectivity

**DOI:** 10.3389/fnhum.2013.00193

**Published:** 2013-05-15

**Authors:** Katja M. Mayer, Quoc C. Vuong

**Affiliations:** ^1^Institute of Neuroscience, Newcastle UniversityNewcastle Upon Tyne, UK; ^2^MPRG Neural mechanisms of human communication, Max Planck Institute for Human Cognitive and Brain SciencesLeipzig, Germany

**Keywords:** multi-featured objects, feature integration, selective attention, psychophysiological interaction, Garner paradigm

## Abstract

Observers can selectively attend to object features that are relevant for a task. However, unattended task-irrelevant features may still be processed and possibly integrated with the attended features. This study investigated the neural mechanisms for processing both task-relevant (attended) and task-irrelevant (unattended) object features. The Garner paradigm was adapted for functional magnetic resonance imaging (fMRI) to test whether specific brain areas process the conjunction of features or whether multiple interacting areas are involved in this form of feature integration. Observers attended to shape, color, or non-rigid motion of novel objects while unattended features changed from trial to trial (change blocks) or remained constant (no-change blocks) during a given block. This block manipulation allowed us to measure the extent to which unattended features affected neural responses which would reflect the extent to which multiple object features are automatically processed. We did not find Garner interference at the behavioral level. However, we designed the experiment to equate performance across block types so that any fMRI results could not be due solely to differences in task difficulty between change and no-change blocks. Attention to specific features localized several areas known to be involved in object processing. No area showed larger responses on change blocks compared to no-change blocks. However, psychophysiological interaction (PPI) analyses revealed that several functionally-localized areas showed significant positive interactions with areas in occipito-temporal and frontal areas that depended on block type. Overall, these findings suggest that both regional responses and functional connectivity are crucial for processing multi-featured objects.

## Introduction

Humans can selectively attend to different features of the same object, such as shape, color, or motion (see Maunsell and Treue, 2006, for a review). At the cortical level, areas that preferentially respond to these separate object features have been identified. For instance, areas in the lateral occipital cortex show preferential responses to intact images of objects as opposed to scrambled images (referred to as the lateral occipital complex, LOC; Malach et al., 1995). Similar feature-preferring areas have been found for color (V4 and V8; McKeefry and Zeki, 1997; Hadjikhani et al., 1998) and motion (area MT; Zeki, 1980; Tootell et al., 1995). It is well established that attention to specific object features can increase the hemodynamic responses in areas that preferentially respond to those features (Corbetta et al., 1990, 1991; Peuskens et al., 2004; Schultz and Lennert, 2009; see Kanwisher and Wojciulik, 2000 for a review).

Thus, selective attention can help observers interact with a complex and dynamic environment by recruiting appropriate neural resources to process object features that are relevant for the task at hand. However, it may still be beneficial to implicitly process or encode information about unattended features because they may become task-relevant under different circumstances. It is therefore important to have neural mechanisms that automatically process features belonging to the same object irrespective of whether they are or are not relevant for the task at hand. This automatic processing can be considered a form of feature integration. The results of several imaging studies suggest that unattended features can still be processed and may even be integrated with attended features (O'Craven et al., 1999; Paradis et al., 2008; Xu, 2010). One possibility is that specific areas may encode the *conjunction* of multiple features. For example, areas along the fusiform gyrus (FG), posterior parietal cortex, and superior temporal sulcus have been shown to respond to feature conjunctions (Kawasaki et al., 2008; Schultz et al., 2008; Cavina-Pratesi et al., 2010). Other studies further suggest that feature-preferring areas such as visual areas, LOC and MT, may also respond to feature conjunctions (Corbetta et al., 1990; Self and Zeki, 2005; Sarkheil et al., 2008; Seymour et al., 2009).

These regional responses are important but they do not necessarily provide a complete picture of feature integration at the neural level as there are also anatomical and functional connections between areas (Felleman and Van Essen, 1991; Hagmann et al., 2008). These connections may, for instance, allow areas to pool information from other areas. This form of feature integration could therefore also occur through the *functional connectivity* between different brain areas, which can be conceptualized as the correlation in the activation time series between brain areas (Friston et al., 1997; Horwitz, 2003; Bingel et al., 2007; Nummenmaa et al., 2010). These connections can further depend on the attentional state of the observer (Friston et al., 1997). Thus in addition to modulating regional responses, selective attention can modulate the functional connectivity between areas.

To date, no study has been designed to test whether functional connectivity may provide a mechanism to integrate multiple object features (in addition to regional responses to the conjunction of features). We used the Garner paradigm to test this possibility. This paradigm has been a powerful method to test whether features are integral or separable at the *behavioral level* (Garner, 1974, 1988; Gottwald and Garner, 1975; see also Cant et al., 2008). With integral features, observers' performance on a task with an attended feature is affected by changes to the unattended feature (i.e., Garner interference). That is, integral features are processed automatically irrespective of whether they are task-relevant or task-irrelevant. By comparison with separable features, observers' performance on the task with the attended feature is unaffected by changes to the unattended feature. Here we applied the *logic* of the Garner paradigm to a feature-attention task to test whether different features are integral or separable at the *neural level*. Applying the logic of the Garner paradigm to neural responses does not mean that we necessarily expect differences between conditions at the behavioral level.

Observers in our study were presented with novel objects which had distinct three-dimensional shape, color, and non-rigid motion. They were instructed to attend to the shape, the color, or the motion and detect changes to the attended feature while ignoring the remaining features in two types of blocks. On *change* blocks, both unattended features varied from trial to trial. By comparison, on *no-change* blocks, both unattended features were held constant which leads to fMRI adaptation (i.e., a decrease in the BOLD response to the unattended object features; Grill-Spector and Malach, 2001). The two block types allowed us to measure whether and how changes to the unattended features affect regional responses and functional connectivity between areas while keeping the task constant (see Sakai, 2008, for a review of possible effects of task sets induced by the different instructions). The Garner interference is typically measured by the difference in speeded response times between change and no-change blocks (e.g., Cant et al., 2008). We have previously shown that observers' performance was affected by changes to unattended features using the same stimuli as the ones used in the present study (Mayer and Vuong, 2012). However, behavioral differences may drive changes in regional responses or functional connectivity between areas. We therefore designed our fMRI experiment so that performance was equated across the two block types (e.g., Nummenmaa et al., 2010). Thus, in contrast to the standard (behavioral) Garner tasks, we do not expect observers' performance to differ on change and no-change blocks. If shape, color, and motion are separable features at the neural level, we predict no differences in regional responses and functional connectivity for change blocks compared to no-change blocks. However, if these features are integral as suggested by previous studies (e.g., O'Craven et al., 1999; Peuskens et al., 2004; Self and Zeki, 2005; Sarkheil et al., 2008; Schultz et al., 2008; Seymour et al., 2009; Cavina-Pratesi et al., 2010), then there are two possible BOLD signatures that parallel the behavioral signature originally reported by Garner and colleagues (Garner, 1974, 1988; Gottwald and Garner, 1975). If feature integration is accomplished by regional responses, then areas that encode the conjunction of features would show larger activation on change blocks compared to no-change blocks (e.g., O'Craven et al., 1999; Paradis et al., 2008; Xu, 2010). If feature integration is accomplished by functional connectivity between areas, then connections between areas would show larger correlations in their activation time series on change blocks compared to no-change blocks.

## Materials and methods

### Participants

Twelve volunteers (4 males, 8 females; age in years: *M* = 31, *SD* = 6; age range: 24–42 years) participated in this study. All participants were naive to the purpose of the study and gave informed consent. They had never seen the stimuli before. Due to a technical failure, behavioral results were only available for 11 out of 12 participants. The study was performed in accordance with the Declaration of Helsinki and approved by the ethics committee of Newcastle University.

### Stimuli and apparatus

A set of 64 novel colored dynamic objects was used (for details see Mayer and Vuong, 2012). Briefly, each object was defined by a combination of one of four shapes (e.g., cylinder or brick), one of four colors (e.g., red or blue), and one of four non-rigid motions (e.g., bending or twisting). Each object subtended approximately 7.7° (height) × 3.8° (width) of visual angle. Examples of the stimuli can be found at: http://www.staff.ncl.ac.uk/q.c.vuong/MayerVuong.html

We used a canon XEED LCD projector (1280 × 1024 pixels) to backproject the visual stimuli onto a projection screen at the foot-end of the scanner. Participants viewed the projection through an angled mirror attached to the head coil approximately 10 cm above their eyes. The experiment was run on a Windows PC using the Psychophysics toolbox version 3 (http://www.psychtoolbox.org; Brainard, 1997; Pelli, 1997) to control the experiment, present the stimuli and record responses. Participants responded via a MR-compatible response pad (LumiTouch™).

### Design

The experiment was set up as a 2 × 3 within-subjects design with block type (change, no-change) and attended feature (shape, color, motion) as repeated measures. A block design was used in which the six experimental conditions were run in separate blocks. The six blocks were presented twice in a single functional run. The 12 blocks were grouped into four sets. Within each set, each attended-feature condition was presented once and one of the two block-type conditions was presented twice. Both the order of the attended-feature conditions and the repeated block type were randomly determined for each set. In addition to the six experimental blocks, there were five 12 s fixation blocks. The fixation blocks were presented at the beginning of each functional run and after each set of three experimental blocks. There were two functional runs each lasting approximately 7 min in the scanning session.

### Procedure

Participants were instructed outside the scanner and ran through a set of practice blocks of the experiment ensuring that every participant practised each experimental condition. In one experimental block, participants watched a sequence of eight objects. They were instructed to attend to one feature (color, shape, or motion) and to perform a 1-back feature matching task on the attended feature (see also Schultz and Lennert, 2009). On change blocks, the objects were selected so that the unattended features were different on consecutive trials (except on target trials; see below). On no-change blocks, the objects were selected so that the unattended features were the same throughout the block. Participants pressed a button on the response pad with the left thumb whenever the attended feature was repeated on consecutive trials (target trials). No response was required on mismatch trials. Therefore, a response was only required on a small set of trials. We did not explicitly ask participants to respond as quickly as possible as we only implemented the behavioral task to ensure that participants attended to the instructed feature for that block. For each participant, the 64 objects were used as equally often as possible. Furthermore across participants, the 64 objects occurred in all six experimental as equally often as possible.

Each experimental block began with a 2 s instruction (the word: “color,” “shape,” or “motion”) presented at the center of the screen, which indicated the attended feature for that block. The instruction was followed by a 0.7 s blank screen. Within a block, eight objects were shown in total (two of them were target objects; see below). Each object was then shown for 2.5 s at the center of the screen, and was followed by a 0.7 s blank screen. The presentation duration was less than a full cycle of motion for the objects. Based on our previous behavioral data (Mayer and Vuong, 2012), this duration was a sufficient amount of time to judge all three features. In total, an experimental block lasted 27.6 s.

There were two target trials in each experimental block. On target trials, the repeated object matched the preceding object on all three features. This was necessary to keep target trials on change and no-change blocks equivalent as the unattended features on a no-changed block were identical throughout the block. Participants were asked to respond when they detected a target trial. A response was counted as correct if it was made during the 2.5 s presentation of the repeated object on a target trial. Misses (i.e., not responding on a target trial) and responses made on any other occasions were counted as errors. No feedback was provided.

### Functional localizers

We used standard functional localizers to identify brain areas that preferentially responded to shape, motion, or color. The shape and color localizers were combined into a single functional run lasting ~7 min. For these localizers, participants viewed blocks of colored images of common objects, phase-scrambled versions of these images, colored Mondrian patches, and grayscale Mondrian patches. Each image was shown for 0.3 s with a 0.4 s blank between images. There were four blocks of each type containing 18 different images each. The block order was counterbalanced and intermixed with nine fixation blocks (12 s). Data for the motion localizer were collected in a separate functional run lasting ~5 min. The localizer contained eight blocks in which a random-dot field expanded and contracted from the center of the screen (15 s) and eight blocks in which the random-dot field remained static (15 s). The dynamic and static blocks alternated (starting with a dynamic block) with fixation blocks (12 s) intermixed between the two main block types.

### Image acquisition

All participants were scanned at the Newcastle Magnetic Resonance Centre. In the scanner, head motion was restrained with foam pads that were placed between the head and the head coil. Anatomical T1-weighted images and functional T2^*^-weighted echo planar images (EPIs) were acquired from a 3 T Philips Intera Achieva MR scanner using a Philips 8-channel receive-only head coil. The high resolution T1-weighted scan (MP-RAGE) consisted of 150 slices and took approximately 5 min to acquire. The field of view (FOV) was 240 × 240 × 180 mm^3^ with a matrix size of 208 × 208 pixels. Each voxel was 1.15 × 1.15 × 1.2 mm^3^ in size. The T2^*^-weighted EPIs consisted of 27 axial slices acquired from the bottom to the top of the head. The parameters of the EPIs were: *TR* = 1.92 s, *TE* = 40 ms, flip angle = 90°. The FOV was 192 × 192 × 107 mm^3^ with a matrix size of 64 × 64 pixels. Each voxel was 3 × 3 × 3 mm^3^ in size, with a 1 mm gap between slices. We use sensitivity encoding (SENSE) with factor = 2 to increase the signal-to-noise ratio of the functional images. For each participant, a total of 414 functional images were acquired (207 in each run). Before each functional run, four “dummy” scans were acquired to allow for equilibration of the T1 signal. The functional localizers were acquired in the same scanning session using T2^*^-weighted EPIs with the same parameters as the experiment.

### fMRI preprocessing

Functional images of the experiment were realigned to the first image of each participant and resliced to correct for head motion. These images were normalized to a standard MNI EPI T2^*^-weighted template with a resampled voxel size of 3 × 3 × 3 mm^3^. They were then spatially smoothed with a 6 mm full-width-at-half-maximum Gaussian kernel to improve the signal-to-noise ratio and to allow for comparisons across participants. To remove low-frequency drifts in the signal, we applied a high-pass filter with a cutoff of 128 s. We also applied an autoregressive model [AR(1)] to estimate serial correlations in the data and adjust degrees of freedom accordingly. The same procedure was applied to the functional localizers.

### fMRI analyses

The preprocessed data were analysed using SPM5 (http://www.fil.ion.ucl.ac.uk/spm; Friston et al., 1994). We used the general linear model (GLM) with a two-step mixed-effects approach. First, a fixed-effects model was used to analyse each participant's data set. Second, a random-effects model was used to analyse the individual datasets at the group level. No additional smoothing of the images was used at the group level.

The design matrix for each participant was constructed as follows. The onset and duration for each of the six experimental conditions (2 block types × 3 attended features; each with a duration of 27.6 s) and the fixation block (12 s) were modeled as boxcar functions. These boxcar functions were then convolved with a canonical hemodynamic response function (HRF), implemented in SPM5 as the difference of two gamma functions. In addition to these regressors of interest, the six movement parameters (roll, yaw, pitch, and three translation terms), the instruction period (2.0 s), and a constant term for each session were included in the design matrix as regressors of no interest. A linear combination of the regressors was fitted to the BOLD signal to estimate the beta weight for each regressor.

For the first-level analysis, contrast images for the main effect of block type, the main effect of attended feature, and the interaction between block type and attended feature were computed from the beta-weight images. For the second-level group analysis, 1-sample *t*-tests of participants' contrast images were conducted at each voxel. We used an initial (uncorrected) threshold of *p* < 0.001 and a minimum cluster size of *k* = 10 voxels (unless otherwise stated). We accepted clusters as significant if *p* < 0.05, FDR-corrected for multiple comparisons at the cluster level.

Similarly, for our functional localizers, we created a design matrix that included block type as our regressors of interest, and the movement parameters and a constant term as regressors of no interests. After fitting this matrix, we computed contrast images for the contrast of interest for each functional localizer (shape: object > scrambled, color: color > grayscale, and motion: dynamic > static). At the second-level group analysis, 1-sample *t*-tests of participants' contrast images were conducted at each voxel. As for the experimental data, we used an initial (uncorrected) threshold of *p* < 0.001 and a minimum cluster size of *k* = 10 voxels.

### PPI analyses

We used psychophysiological interaction (PPI) analyses (Friston et al., 1997) to identify target areas which show changes in functional connectivity between seed and target areas as a function of block type. For each PPI analysis, a design matrix was constructed for each participant that included the activation time series from a seed area (i.e., the physiological variable), the boxcar time series for the two block types convolved with the canonical HRF (i.e., the psychological variable), and the product between the two time series (i.e., the PPI). The activation time series was computed by deconvolving the first eigenvariate of the BOLD time series from all voxels of the seed area to estimate changes in neural activity in that area (Gitelman et al., 2003). For the psychological variable, change blocks were coded as 1 and no-change blocks were coded as −1. We also included the six movement parameters and a constant term for each session in the design matrix as regressors of no interest. As with the whole-brain analyses, we first estimated regressor beta weights for each participant. We then submitted the participants' beta-weight image for the PPI regressor to a 1-sample *t*-test against zero.

## Results

### Behavioral results

For accuracy, there was no main effect of attended feature or block type, and no interactions between these two factors (all *p*s > 0.11). For response times, there was only a main effect of attended feature [*F*_(2, 20)_ = 35.86; *p* < 0.001; η^2^_*p*_ = 0.78; all remaining *p*s > 0.14]. Tukey's *post-hoc* tests revealed that response times were quicker on attention to color trials compared to the other two attend-feature conditions, and that response times were quicker on attention to shape trials compared to attention to motion trials (*p*s < 0.05). We did not find Garner interference at the behavioral level (Garner, 1974, 1988; Gottwald and Garner, 1975): there were no behavioral differences between change and no-change blocks. However, the experiment was designed to equate performance on these two block types as it has been shown that response times can affect both regional responses and functional connectivity (e.g., Ganel et al., 2005; Prado and Weissman, 2011).

### fMRI results: functional localizers

Table 1 shows the significant cortical areas for the different localizers. For the shape localizer, we focused on the two areas along the left and right lateral occipital cortex and the FG (e.g., Grill-Spector et al., 2001) for the subsequent overlap calculations although we report all significant areas in Table 1. The peak voxel in the left hemisphere was in the cerebellum (as reported by the WFU PickAtlas; http://fmri.wfubmc.edu/software/PickAtlas; Maldjian et al., 2003). We therefore reported a peak in cortex which was a sub-cluster of the area based on the coordinate of the peak voxel in the right hemisphere.

**Table 1:** **Brain areas activated by the shape, motion, and color localizers**.

**Brain area**	**MNI**			***k***	***z***
	***x***	***y***	***z***		
**SHAPE: OBJECTS > SCRAMBLED**					
PG	30	−45	−12	962	5.07
PG	−15	−9	−18	12	3.72
FG^*^	−30	−57	−12	1053	5.06
Cun	−6	−69	6	32	4.02
Cun	15	−75	3	22	3.98
Cun	12	−78	9	15	3.63
SOG	36	−84	30	15	3.44
**MOTION: MOTION > SCRAMBLED**					
IOG	−27	−93	−6	239	4.99
IOG	39	−72	−9	56	3.98
Cun	27	−99	−6	245	4.80
**COLOR: COLOR > GRAYSCALE**					
LG	18	−90	0	58	4.08
FG	−33	−75	−18	27	4.22
FG	−36	−54	−21	13	3.58
MOG	33	−78	−15	51	4.25

*p < 0.05, FDR-corrected at the cluster level*.

*IOG, inferior occipital gyrus; Cun, cuneus; PG, parahippocampal gyrus; LG, lingual gyrus; FG, fusiform gyrus; MOG, middle occipital gyrus; SOG, superior occipital gyrus. Cluster size (k) for a given localizer includes voxels that overlapped with other localizers*.

* *The global maximum of this cluster is in the cerebellum, therefore we report the local maximum in the FG*.

**Figure 1:**
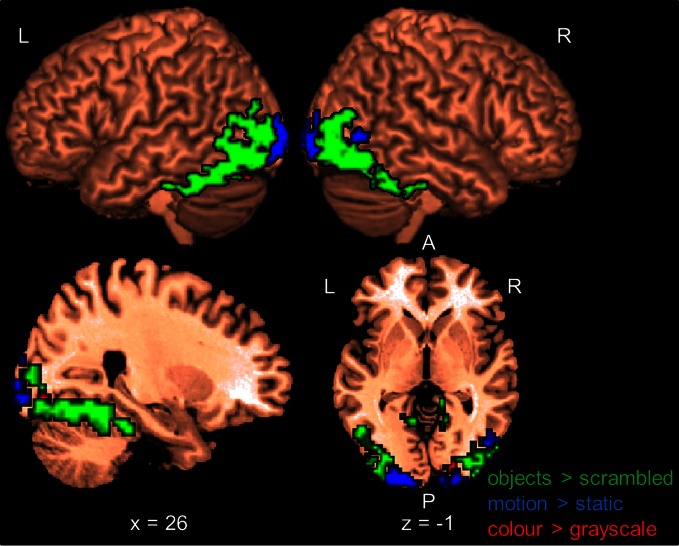
**Results of the functional shape, motion, and color localizers for the whole-brain group-analysis (*N* = 12).** Areas are significant at *p* < 0.05, FDR-corrected at the cluster level for multiple comparisons across the whole-brain. Only voxels that were uniquely activated by one localizer are shown. L, left; R, right; A, anterior; P, posterior. Coordinates are in MNI space.

Within each hemisphere, the areas activated by the localizers overlapped with each other. However, we needed masks of the localizers that did not overlap with each other to determine whether the areas identified by our whole-brain and PPI analyses in the main study overlapped uniquely with functionally localized shape-, motion- and color-selective areas. Thus, voxels that were activated by more than one localizer were excluded when computing overlap (see below). Figure 1 shows only voxels that were uniquely activated by one of the localizers.

### fMRI results

#### Attention to shape (S), color (C), and motion (M)

**Figure 2:**
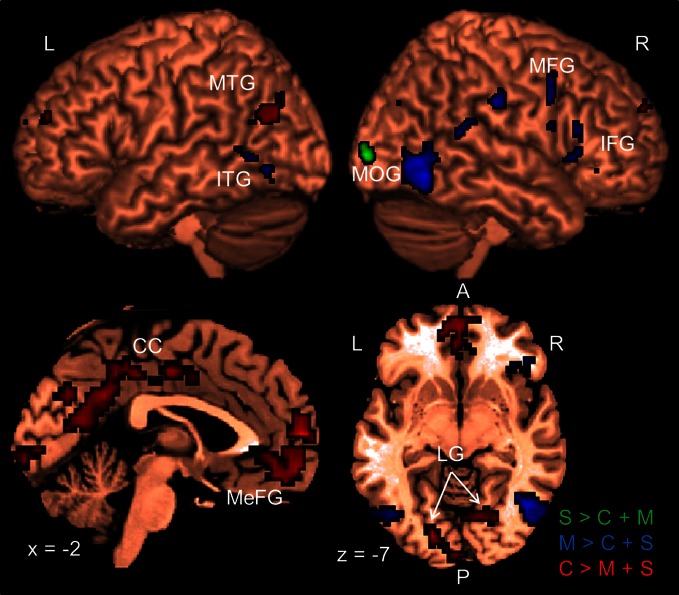
**Results of the whole-brain group-analysis (*N* = 12) for the Garner task averaged across change and no-change blocks.** Areas are significant at *p* < 0.05, FDR-corrected at the cluster level for multiple comparisons across the whole-brain. Slice numbers are in MNI space. L, left; R, right; A, anterior; P, posterior; S, shape; C, color, M, motion; MTG, middle temporal gyrus; ITG, inferior temporal gyrus; MOG, middle occipital gyrus; MFG, middle frontal gyrus; IFG, inferior frontal gyrus; CC, cingulate cortex; MeFG, medial frontal gyrus; LG, lingual gyrus.

Following previous work (e.g., Corbetta et al., 1990; Peuskens et al., 2004), we identified areas that preferentially responded to the attended object feature by contrasting blocks in which a feature was attended with blocks in which the other two features were attended. Activated areas were labeled with the WFU Pickatlas (Maldjian et al., 2003). Figure 2 and Table 2 present areas which showed larger activation for the attended features with respect to the other two features. When participants attended to shape (S > C + M), only one area in the right MOG was significant.

**Table 2:** **Brain areas which showed larger responses for attention to a feature relative to attention to the remaining features**.

**Brain area**	**MNI**			***k***	***z***
	***x***	***y***	***z***		
**ATTENTION SHAPE > ATTENTION MOTION/COLOR**					
MOG	33	−96	3	18	4.18
**ATTENTION MOTION > ATTENTION SHAPE/COLOR**					
MOG	48	−63	−9	198	4.80
ITG	−42	−69	−6	90	4.10
STG	66	−45	15	28	4.06
MFG	27	0	57	33	3.98
MFG	−30	−3	51	37	3.85
IFG1	54	21	3	33	3.92
IFG2	45	33	−6	24	3.98
IFG3	39	6	36	45	4.13
IFG4	57	9	18	16	3.55
IPL	39	−33	39	22	3.84
PCG	63	−24	33	40	4.41
Cerebellum	33	−72	−27	123	4.57
Cerebellum	−36	60	−30	167	4.69
**ATTENTION COLOR > ATTENTION SHAPE/MOTION**					
LG^*^	24	−72	−6	39	3.68
LG	−18	−81	−9	35	4.34
Cun	−3	−96	−6	21	3.75
Cun	15	−90	27	21	3.40
MTG	−48	−75	27	82	3.97
ITG	−33	−30	−12	16	3.88
PHG	33	−15	−24	17	4.28
MFG	−24	51	21	29	3.79
MeFG	3	63	9	307	4.64
PreCun	−3	−72	33	19	3.84
CC	6	−12	45	59	4.23
CC	6	−36	39	315	4.26

*p < 0.05, FDR-corrected at the cluster level*.

*k, number of voxels in the cluster; MOG, middle occipital gyrus; ITG, inferior temporal gyrus; MFG, middle frontal gyrus; IFG, inferior frontal gyrus; IPL, inferior parietal lobule; STG, superior temporal gyrus; MeFG, medial frontal gyrus; Cun, cuneus; PreCun, precuneus; MTG, middle temporal gyrus; PHG, parahippocampal gyrus; CC, cingulate cortex; PCG, postcentral gyrus; LG, lingual gyrus*.

* *This peak voxel is not allocated to any anatomical landmark in the WFU pickatlas. The label of a voxel adjacent to the peak is reported*.

To test whether the activated area was consistent with the areas that the localizer identified as shape-areas we divided the number of voxels contained in a localizer mask by the total number of voxels of the area. The cluster activated when participants attended to shape did not overlap with the LOC localizer and only 11% of the area overlapped with the MT localizer (Table 3).

**Table 3:** **Brain areas from the whole-brain analyses that overlapped with the functional localizers for shape, motion, and color**.

**Brain area**	**MNI**			**Proportion in localizer**		
	***x***	***y***	***z***	**Shape**	**Motion**	**Color**
**ATTENTION SHAPE > ATTENTION MOTION/COLOR**						
MOG	33	−96	3	0	0.11	0
**ATTENTION MOTION > ATTENTION SHAPE/COLOR**						
MOG	48	−63	−9	0.27	0.08	0
ITG	−42	−69	−6	0.38	0	0
**ATTENTION COLOR > ATTENTION SHAPE/MOTION**						
LG^*^	24	−72	−6	0.08	0	0
Cun	−3	−96	−6	0	0.10	0
ITG	−33	−30	−12	0.25	0	0

*MOG, middle occipital gyrus; ITG, inferior temporal gyrus; LG, lingual gyrus; Cun, cuneus*.

* *This peak voxel is not allocated to any anatomical landmark in the WFU pickatlas. The label of a voxel adjacent to the peak is reported*.

Previous studies have reported activation in temporal, parietal, and frontal areas when observers processed an object's shape (Murray et al., 2003; Peuskens et al., 2004; Paradis et al., 2008; Schultz et al., 2008). To further explore our results, we lowered the initial threshold to *p* < 0.01 and accepted areas whose peak voxel had an uncorrected *p* < 0.001. As shown in Table 4, there was a trend that attention to shape activated areas in the right occipital cortex (one at the posterior part and one more ventrally at the occipito-temporal junction), in the left ventral occipito-temporal and in parietal cortices (Peuskens et al., 2004; Schultz et al., 2008).

**Table 4:** **Additional brain areas which showed larger responses for attention to shape relative to attention to motion and color**.

**Brain area**	**MNI**			***k***	***z***
	***x***	***y***	***z***		
**ATTENTION SHAPE > ATTENTION MOTION/COLOR**					
IOG	−33	−96	−9	21	3.36
IOG	−45	−57	−9	28	3.21
ITG	51	−63	−15	24	3.24
PreCun	30	−51	48	48	3.42
IPL	−42	−42	39	27	2.87

*p < 0.001, uncorrected at the peak voxel level*.

*k, number of voxels in the cluster; IOG, inferior occipital gyrus; ITG, inferior temporal gyrus; PreCun, precuneus; IPL, inferior parietal lobule*.

Several areas showed larger activation when participants attended to motion relative to when they attended to the other features (M > C + S). An area in the posterior temporal cortex extending to lateral occipital areas was activated in each hemisphere. These areas overlapped with the shape localizer (27 and 38%) and one of them overlapped slightly with the motion localizer (8%). Consistent with previous work (e.g., Peuskens et al., 2004; Paradis et al., 2008; Schultz et al., 2008), areas were also found in the parietal and lateral frontal cortex. Additionally, we found significant activation in the postcentral gyrus, the superior temporal gyrus (e.g., Schultz et al., 2008) and the cerebellum.

Attention to color (C > M + S) also activated several areas across the brain. There were significant bilateral areas along the collateral sulcus (CoS) and the cuneus (Cun). These areas are consistent with previously reported areas that were activated when observers processed the color of static objects (e.g., Cant and Goodale, 2007; Cant et al., 2008; Cavina-Pratesi et al., 2010). However, these areas did not overlap with the color localizer but three of them overlapped slightly with the shape and motion localizers (8–25%). Activations were also found in the temporal and frontal cortices. Previous studies have reported activation in frontal areas when observers attended to color (e.g., Cavina-Pratesi et al., 2010). We also found large areas in the cingulate cortex (CC) when observers attended color that have not been previously reported.

#### Change vs. no-change blocks

We also tested for cortical areas with larger responses during change blocks compared to no-change blocks. We expected very little adaptation to occur when both unattended features varied from trial to trial because of the drastic visual changes that occurred between trials. By comparison, we expected fMRI adaptation to occur when the unattended features were held constant within a block (Grill-Spector and Malach, 2001). Surprisingly, there were no significant areas localized by this contrast. Furthermore, no areas showed a significant interaction between block type and attended features.

### PPI results

We ran PPI analyses to test whether the functional connectivity between areas depended on block type. All 24 areas from the whole-brain analyses were used as seed areas in the PPI analyses (Table 2). To control for unequal cluster sizes, we used spheres with a 6 mm radius centered on the peak voxel of each area. Thus, each seed area consisted of 33 voxels except for the left cuneus (25 voxels) from the attend-color condition, and the right MOG (26 voxels) from the attend-shape condition. This was due to the lateral location of the peak voxel for these areas which led to an overlap between the sphere and extra-cortical space. Although we interpret all significant PPIs at our *p* < 0.05, FDR-corrected at the cluster-level threshold, we further indicate those PPIs that also remain significant after a Bonferroni correction (see note in Table 5).

**Table 5:** **Target brain areas which showed a significant positive change in functional connectivity between seed and target areas on change blocks relative to no-change blocks**.

**Brain area**	**MNI**			***k***	***z***	**Regression coefficient**	
	***x***	***y***	***z***			**Change**	**No-change**
**RIGHT MOG^†^ ATTENTION SHAPE > ATTENTION MOTION/COLOR**							
ITG	−54	−69	−3	59	3.50	0.71 (0.13)	0.34 (0.13)
**RIGHT MOG^†^ ATTENTION MOTION > ATTENTION SHAPE/COLOR**							
SOG	−30	−75	24	67	4.26	0.27 (0.05)	0.21 (0.11)
MOG	−45	−66	−9	92	4.16^*^	0.61 (0.06)	0.43 (0.08)
MeFG	−3	27	39	66	3.73	0.31 (0.12)	0.21 (0.10)
**LEFT ITG^†^ ATTENTION MOTION > ATTENTION SHAPE/COLOR**							
CC	−6	21	42	124	3.76^*^	0.19 (0.07)	0.12 (0.03)
**RIGHT IFG4^†^ ATTENTION MOTION > ATTENTION SHAPE/COLOR**							
IFG	30	30	−21	57	3.48	0.08 (0.06)	−0.06 (0.05)
**RIGHT LG^†^ ATTENTION COLOR > ATTENTION SHAPE/MOTION**							
MOG	48	−66	−15	90	3.64	0.20 (0.10)	0.06 (0.07)
Cun	27	−72	27	84	3.57	0.16 (0.06)	−0.01 (0.05)
MeFG	12	18	48	120	4.05^*^	0.10 (0.06)	0.05 (0.03)
SMG	57	−48	33	58	3.64	0.27 (0.05)	0.18 (0.06)
**LEFT LG^†^ ATTENTION COLOR > ATTENTION SHAPE/MOTION**							
IFG	−51	15	0	70	3.29	0.31 (0.10)	0.18 (0.11)
**LEFT CUN^†^ ATTENTION COLOR > ATTENTION SHAPE/MOTION**							
CC	−6	9	45	81	3.64	0.13 (0.05)	0.07 (0.06)
**RIGHT PHG^†^ ATTENTION COLOR > ATTENTION SHAPE/MOTION**							
PreCun	24	−66	21	73	3.93	0.13 (0.05)	0.09 (0.05)
**LEFT ITG^†^ ATTENTION COLOR > ATTENTION SHAPE/MOTION**							
CC	0	48	12	487	3.88^*^	0.14 (0.09)	0.08 (0.10)
AG	−51	−66	33	103	3.55^*^	0.15 (0.05)	0.10 (0.09)

*p < 0.05, FDR-corrected for multiple comparisons at the cluster level*.

* *p < 0.002 (p-value Bonferroni corrected for 24 seed regions)*.

*k, number of voxels in the cluster*.

† *Areas used as seed regions for the psychophysiological interaction analysis (see Table 2). The regression coefficient for each seed-target pair and block type was computed separately for each participant and then averaged. The standard error of the mean is shown in parentheses. AG, angular gyrus; SOG, superior occipital gyrus; ITG, inferior temporal gyrus; MOG, middle occipital gyrus; IFG, inferior frontal gyurus; MeFG, medial frontal gyrus; CC, cingulate cortex; Cun, cuneus; SMG, supramarginal gyrus; PreCun, precuneus; LG, lingual gyrus; PHG, parahippocampal gyrus*.

**Figure 3:**
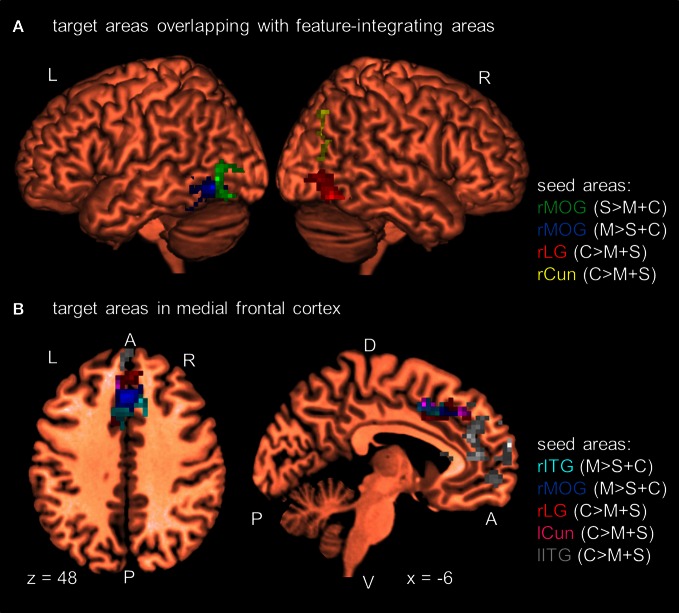
**Results of the PPI analyses at the group level (*N* = 12).** Seed areas refer to areas activated in the GLM (Table 2). Areas are significant at *p* < 0.05, FDR-corrected at the cluster level for multiple comparisons across the whole-brain. Coordinates are in MNI space. L, left; R, right; A, anterior; P, posterior; C, attention to color; S, attention to shape; M, attention to motion; rITG, right inferior temporal gyrus; rCun, right cuneus; rMOG, right middle occipital gyrus; rLG, right lingual gyrus; lITG, left inferior temporal gyrus; lCun, left cuneus. For clarity only a selection of target areas are shown (see Table 5). **(A)** Target areas that overlapped with MT and LOC as localized by our MT- and LOC-localizer. **(B)** Target areas that cluster in medial frontal cortex.

As shown in Figure 3 and Table 5, the PPI analyses identified several target areas that showed a positive change in the functional connectivity between the seed and the target areas on change blocks relative to no-change blocks (i.e., a significant positive PPI). These target areas clustered in the posterior and anterior parts of the brain. We further regressed the activity of the target areas onto the BOLD times series of the seed areas to determine the nature of this positive interaction (e.g., Nummenmaa et al., 2010). To do so, we first shifted all time series by 3 TRs (~6 s) to account for the hemodynamic delay and then categorized time points as a change block or no-change block. We then computed the participant-wise regression coefficient for each seed-target pair separately for the change time points and no-change time points. Nearly all pairs had a positive regression coefficient when averaged across participants (Table 5). The coefficients were also positive when averaged across all seed-target pairs (change block: *M* = 0.26, *SE* = 0.04; no-change block: *M* = 0.15, *SE* = 0.03).

In addition to these findings, we found that four seed areas (attend-motion: right MOG and left ITG; attend-shape: right MOG; attend-color: right CoS) showed significant PPIs with target areas that overlapped with the shape localizer (37–100%; Table 6; see also Self and Zeki, 2005). The left Cun seed area also showed a significant PPI with a target area that completely overlapped with the motion localizer (Sarkheil et al., 2008). Shape- and motion-preferring regions have previously been shown to integrate multiple object features (Self and Zeki, 2005; Sarkheil et al., 2008). Moreover, five of the nine seed areas showed significant PPIs with *both* posterior and anterior targets. Thus, although there were no changes in regional responses as a function of block type (no main effect of block type and no interaction between block type and attention condition), there were changes to the functional connectivity between seed and target areas.

**Table 6:** **Target brain areas from the PPI analyses that overlapped with the functional localizers for shape (LOC), motion (MT), and color (V4)**.

**Brain area**	**MNI**			**Proportion in localizer**		
	***x***	***y***	***z***	**Shape**	**Motion**	**Color**
**RIGHT MOG^†^ ATTENTION SHAPE > ATTENTION MOTION/COLOR**						
ITG	−54	−69	−3	0.56	0	0
**RIGHT MOG^†^ ATTENTION MOTION > ATTENTION SHAPE/COLOR**						
MOG	−45	−66	−9	0.37	0	0
**RIGHT LG^†^ ATTENTION COLOR > ATTENTION SHAPE/MOTION**						
MOG	48	−66	−15	0.62	0.08	0
Cun	27	−72	27	0.02	0	0

*ITG, inferior temporal gyrus; MOG, middle occipital gyrus; Cun, cuneus; LG, lingual gyrus*.

* *Seed areas used for the psychophysiological interaction analysis (see Table 2)*.

## Discussion

In the present study, we investigated the neural signature underlying the automatic processing of object features. We adapted a Garner paradigm for functional imaging (Garner, 1974, 1988; Gottwald and Garner, 1975; see also Ganel et al., 2005) in which observers either attended to the shape, color or non-rigid motion of novel colored dynamic objects while the unattended features changed or remained constant. Surprisingly we found that changes to unattended features did not modulate regional responses, despite large trial-by-trial changes to the visual stimulus. Rather, we found that changes to unattended features modulated the functional connectivity between areas.

Consistent with a large body of work, we found that selective attention to features increased regional responses in areas involved in processing those features (e.g., Corbetta et al., 1990, 1991; Paradis et al., 2000, 2008; Murray et al., 2003; Peuskens et al., 2004; Self and Zeki, 2005; Cant and Goodale, 2007; Cant et al., 2008; Sarkheil et al., 2008; Schultz et al., 2008; Seymour et al., 2009; Cavina-Pratesi et al., 2010). This important replication demonstrated that we could identify feature-preferring areas with our paradigm despite using a simple task. The areas activated by selective attention generally did not overlap with areas identified by the functional localizers but they were spatially close. The differences in spatial location may reflect the fact that more complex stimuli were used in the Garner paradigm compared to the stimuli used in the localizer runs. Another possibility is that selective attention to an object feature may shift the spatial locus of activation. This is an interesting possibility that could be explored in future studies.

Additionally, we found larger activation in CC when observers attended to color compared to when they attended to shape or motion. Activations in these areas were not found in previous studies on color processing (Self and Zeki, 2005; Cant and Goodale, 2007; Cant et al., 2008, 2009; Seymour et al., 2009; Cavina-Pratesi et al., 2010) but that may be due to the nature of the task used in present study compared to those used in previous studies (see Mayer and Vuong, 2012). The area we found in CC can be divided into an anterior and posterior part (ACC and PCC, respectively). From a theoretical perspective, activations in the ACC and PCC may reflect different strategies for processing shape, color, and motion. These areas may also be involved in how attention is allocated to different features (Schultz and Lennert, 2009). The ACC has been shown to be involved in performance monitoring on a Stroop task, for instance (MacDonald et al., 2000; see Botvinick et al., 2004, for a review). This finding is consistent with the ACC's role in monitoring performance more carefully on mismatched trials. Observers in our study responded more quickly when they attended to color compared to when they attended to shape or motion, suggesting that they may be monitoring their performance differently when they attended to color than when they attended to shape or motion. For example, they may have been able to focus on local “patches” to discriminate color but had to focus on global shape or motion to discriminate the other two features (see Peuskens et al., 2004). The PCC activation seems to further support the possibility that observers used different processing strategies for color than for shape or motion. The PCC is part of the default-mode network (Buckner et al., 2008) and has been found to show *deactivations* when observers attend to external events (i.e., during an explicit task) relative to a resting state. In particular, larger activation of the PCC has been observed for “easy” tasks compared to “hard” tasks, presumably because “hard” tasks require more external monitoring (e.g., Leech et al., 2011). That said, it may not be straightforward to interpret ACC and PCC activation as a consequence of task difficulty. In our study, participants showed quickest responses for color. Accuracy, however, did not differ across features. Furthermore, Gilbert et al. (2012) suggested that measures such as response times and accuracy may not necessarily be good predictors for deactivations of areas that are part of the default-mode network. Future work will be needed to understand the precise role of the ACC and PCC in selective attention and how these roles may relate to different processing strategies for different object features.

Selective attention had a robust effect on the regional responses of several brain areas in our study. Previous research indicated that unattended visual features also elicit distinct BOLD responses (O'Craven et al., 1999; Peuskens et al., 2004; Ganel et al., 2005; Paradis et al., 2008). We therefore expected differences in regional responses in some brain areas when unattended features varied or remain constant because of adaptation (Grill-Spector and Malach, 2001). However, we found that changes to unattended object features did not affect regional responses in any areas relative to when these features remained constant. We also found no areas which showed differential activations between these two conditions when we focused our analyses on each attended feature separately.

Although we did not find overall changes in BOLD responses depending on whether unattended features changed or not, we found that unattended features affected neural responses between areas using PPI analyses (Friston et al., 1997). First and most importantly, our PPI analyses identified target areas that showed an increased functional connectivity between seed and target areas. We re-emphasize that we designed the experiment to ensure that performance was equated across change and no-change blocks (e.g., Nummenmaa et al., 2010). Observers were not instructed to respond quickly. They responded only on trials on which the attended feature changed but not on other trials leading to two responses per eight trials. We have previously shown that changes to unattended features can affect performance using the same stimuli (Mayer and Vuong, 2012). Thus, the modulation of functional connectivity by changes to unattended features is unlikely due to differences in performance (cf., Ganel et al., 2005). Rather, the functional connectivity between areas suggests that observers continued to automatically process the unattended features at the neural level. Our finding is consistent with Friston et al.'s (1997) finding that attention to motion increased functional connectivity between motion-selective areas when observers attended to the motion of moving dots relative to when they passively viewed the same stimuli. Our finding is also consistent with recent work by Prado and Weissman (2011). They found that trial-by-trial variations in response times were correlated with variations in functional connectivity between middle occipital gyrus and FG. They also showed that observers' covert spatial attention influenced the relationship between response times and functional connectivity but did not influence regional responses in these areas. These studies highlight the importance of functional connectivity even if there are no differences in regional responses across conditions.

Second, increases in functional connectivity were predominantly found between occipito-temporal seed areas and their target areas (i.e., 8 occipito-temporal areas out of 24 seed areas). Only one seed area in the right IFG showed a significant interaction with a target area in the right MFG. Furthermore, six of the occipito-temporal seed areas showed a significant increase in functional connectivity between seeds and occipito-temporal target areas which overlapped with the LOC and MT localizers. These six target areas (lingual gyrus: LG, ITG, and MOG) were located posteriorly in areas of visual cortex that are known to process basic visual features such as color (McKeefry and Zeki, 1997), motion (Tootell et al., 1995), or shape (Malach et al., 1995). Importantly, shape- and motion-preferring areas have previously been found to integrate color and motion (LOC; see Self and Zeki, 2005) and shape and motion (MT; see Sarkheil et al., 2008). The increase in functional connectivity found in our study may indicate that these areas also pool information about unattended features processed in other areas, such as LG, as a form of feature integration. Alternatively, the increase in functional connectivity may indicate that shape- and motion-preferring areas suppress attentional resources automatically recruited by the unattended features and/or strengthen responses in areas processing the attended feature in the face of distraction by the unattended features. Future work is needed to tease apart these alternatives.

Lastly, we found that several occipito-temporal seed areas (5 out of 7) showed significant interactions with *both* posterior and anterior cortical target areas. The anterior target areas included those in the ACC, medial frontal gyrus, and IFG. These frontal areas have been implicated in monitoring external events or gauging task difficulty (e.g., MacDonald et al., 2000; Weissman et al., 2005; Leech et al., 2011; Gilbert et al., 2012). For example, MacDonald et al. (2000) found that the ACC monitored mismatches between color labels and their printed color. Furthermore, Weissman et al. (2005) showed that the activity in the dorsal parts of the ACC predicted the amount of behavioral interference by distracting events on a task in which participants had to attend to local or global features of a stimulus. Frontal areas have also been implicated as part of an attentional network which provides top-down cognitive control of a task (e.g., Cabeza et al., 2003). The significant interactions between seed areas and the frontal target areas observed in our study suggest that there may be some top-down control of posterior areas to monitor the larger amount of changing visual information on change blocks compared to no-change blocks. However, this interpretation is speculative as we need to be cautious about interpreting the direction of influence from PPI analyses (Gitelman et al., 2003; Nummenmaa et al., 2010). Overall, our results highlight the importance of functional connections between anterior task-monitoring areas and posterior feature-preferring areas and between posterior areas for processing multi-feature objects. These connections are particularly important when observers are attending to some features while ignoring others.

In our study, observers were instructed to attend to different features. This instruction may have induced different task sets which may have modulated both regional responses and interactions between areas (see Sakai, 2008, for a review). Sakai and Passingham (2003) found that the correlation in activity between anterior prefrontal areas and posterior prefrontal areas (e.g., superior frontal sulcus and IFG) depended on whether observers were instructed to remember items in the order they were presented or in reversed order. Interestingly, these interactions between prefrontal areas were observed during a delay period before the task began and were sustained throughout the task. Task set rather than selective attention may have partly driven some of the BOLD responses we observed in our study. However, the modulation of functional connectivity between areas cannot be driven solely by task set as the key comparison was whether unattended features varied or remained constant for a given attended feature (observers had to attend to shape, motion, and color during both change and no-change blocks).

Previous studies have identified several areas that respond selectively to the conjunction of features. These feature-conjunction areas have been identified along the ventral and dorsal processing pathways. We did not find any areas that responded selectively to the conjunction of multiple object features in our study (though caution is warranted for null findings). However, our results complement the results of these previous studies rather than contradict them. It is most likely the case that there are complementary mechanisms to automatically process features that belong to the same object. These mechanisms can work alone or in combination. Importantly, they allow observers to adaptively select those object features that are the most effective for the task at hand, while at the same time allowing them to encode or process unattended features that may become task-relevant under different circumstances.

### Conflict of interest statement

The authors declare that the research was conducted in the absence of any commercial or financial relationships that could be construed as a potential conflict of interest.
